# Alemtuzumab‐Induced Graves’ Disease Complicated by Pretibial Myxoedema: A Case Report

**DOI:** 10.1155/crie/5617569

**Published:** 2026-08-05

**Authors:** Aongus O’Brolchain, Elham Reda

**Affiliations:** ^1^ Department of Endocrinology, Gold Coast University Hospital, Southport, Queensland, Australia, qld.gov.au; ^2^ School of Medicine and Dentistry, Griffith University, Gold Coast, Queensland, Australia, griffith.edu.au

**Keywords:** alemtuzumab, Graves, pretibial myxoedema

## Abstract

**Background:**

Alemtuzumab, a monoclonal antibody targeting CD52, is associated with a high incidence of secondary autoimmune disease, most commonly thyroid dysfunction. Alemtuzumab‐induced Graves’ disease (AIGD) typically follows a relapsing course and may exhibit atypical or severe phenotypes during immune reconstitution (IR).

**Case Presentation:**

We report a case of severe thyrotoxicosis following alemtuzumab therapy for multiple sclerosis, complicated by the development of thyroid eye disease (TED) and pretibial myxoedema (PTM). Despite escalation of antithyroid therapy, biochemical and clinical control remained suboptimal. Radioiodine therapy was not pursued due to the presence of established orbitopathy. Given persistent severe disease and extrathyroidal manifestations, definitive management with total thyroidectomy was undertaken.

**Results:**

Thyroidectomy resulted in rapid biochemical control of thyrotoxicosis. thyroid‐stimulating hormone‐receptor antibody (TRAb) remained markedly elevated after thyroidectomy, indicating persistent autoimmune activity despite removal of the thyroid gland.

**Conclusion:**

This case highlights an aggressive and uncommon presentation of AIGD characterised by severe thyrotoxicosis, orbitopathy and PTM. Clinicians should recognise the potential for atypical and refractory disease following alemtuzumab therapy. In selected cases, early consideration of definitive therapy is warranted, particularly when radioiodine is considered unsuitable.

## 1. Introduction

Alemtuzumab is a humanised monoclonal antibody targeting CD52 that induces rapid, profound lymphocyte depletion [1]. In relapsing‐remitting multiple sclerosis (RRMS), it improves relapse rates and disability outcomes compared with interferon‐β, but secondary autoimmunity emerges in up to one‐third of patients [2]. Autoimmune thyroid disease (AITD) is the most frequent phenotype (predominantly Graves’ disease, which occurs in up to 40% of treated patients [3]). Thyroid dysfunction typically arises 6–48 months after treatment and may persist or fluctuate with immune reconstitution (IR) [4]. Extrathyroidal features of Graves’ disease—including orbitopathy and, less commonly, pretibial myxoedema (PTM; thyroid dermopathy)—add morbidity and require coordinated care. We present a case of alemtuzumab‐induced Graves’ disease (AIGD) complicated by PTM and discuss mechanisms, incidence and management considerations.

## 2. Case Presentation

A 47‐year‐old woman with RRMS received two cycles of alemtuzumab 12 months apart in 2016 and 2017. She was diagnosed with MS in 2007 after she presented with optic neuritis and had previously been treated with glatiramer and interferon. She was confirmed to be euthyroid prior to commencing treatment (Table 1). In December 2018, she developed palpitations, heat intolerance and increased sweating. Blood tests revealed suppressed thyroid‐stimulating hormone (TSH; <0.05 mIU/L, reference range: 0.55–4.78 mIU/L), elevated free thyroxine (fT4; 35 pmol/L, reference range: 10–20 pmol/L), elevated free triiodothyronine (fT3; 14 pmol/L, reference range: 2.8–6.8) and positive TSH‐receptor antibody (TRAb; 7.5 IU/L, reference range: <1.8 IU/L), consistent with AIGD. She was referred to the endocrinology clinic and carbimazole (CBZ) 10 mg twice daily was commenced in February 2019. The patient had no other contributory past medical history and no family history of thyroid disease.

**Table 1:** Thyroid function tests, thyroid‐stimulating immunoglobulin levels and carbimazole dosing over time.

Date	TSH (mIU/L)	Free T4 (pmol/L)	Free T3 (pmol/L)	TSI (IU/L)	TRAb (IU/L)	Carbimazole dose
Reference range	0.55–4.78	10–20	2.8–6.8	<0.55	<1.8	–
June 2016	2.0	–	–	–	—	–
December 2018	<0.02	35	15	–	7.5	–
March 2019	<0.05	–	–	–	—	10 mg twice daily
May 2019	<0.02	35	15	–	—	20 mg twice daily
September 2019	<0.02	32	16.6	–	—	15 mg three times daily
December 2019	<0.05	32	15.7	33	—	15 mg three times daily
January 2020	<0.02	16	7.9	–	—	10 mg twice daily
September 2020	<0.02	22	13	37	—	10 mg twice daily
April 2021	<0.02	14	8.3	19	—	20 mg twice daily
November 2021	<0.02	38	18.6	–	—	20 mg three times daily
December 2021	<0.02	21	12.2	–	—	10 mg twice daily
Early 2022	<0.02	28	14.9	–	—	10 mg three times daily
April 2022	<0.02	37	20.9	23	—	20 mg morning, 10 mg midday, 20 mg evening
June 2022	<0.02	23	11.7	9.5	—	20 mg three times daily
August 2022	<0.02	18	10.0	–	—	20 mg three times daily
November 2022	<0.02	18	10.8	14	—	25 mg three times daily plus Lugol’s iodine
January 2023	–	–	–	–	—	Thyroidectomy
June 2024	0.7	14.8	3.3	—	160	–
February 2026	1.50	14.3	3.3	—	80	–

*Note:* Carbimazole doses are presented as prescribed at each time point. Missing data are indicated by dashes ‘–’.

Abbreviations: TRAb, TSH‐receptor antibodies; TSI, thyroid‐stimulating immunoglobulin.

On examination, a diffuse goitre was visible on inspection (WHO grade 2). There was a mild tremor but no evidence of thyroid eye disease (TED), acropachy or PTM. By December 2019, she remained thyrotoxic with high thyroid‐stimulating immunoglobulin (TSI) titres (33 IU/L, fT4 32 pmol/L and fT3 15.7 pmol/L), despite increasing CBZ to 15 mg three times daily. During this period, her weight dropped from 74 to 64 kg. Attempts to wean the dose of CBZ were unsuccessful, with sustained disease activity (Table 1). The chronology of alemtuzumab exposure, development of thyroid disease, extrathyroidal manifestations, treatment and subsequent follow‐up is summarised in Table 2.

**Table 2:** Clinical timeline of alemtuzumab exposure, thyroid disease and treatment.

Date	Clinical event
June 2016	Euthyroid before treatment; first cycle of alemtuzumab administered for relapsing‐remitting multiple sclerosis
2017	Second cycle of alemtuzumab administered 12 months after the first cycle
December 2018	Developed palpitations, heat intolerance and increased sweating; biochemical testing demonstrated thyrotoxicosis with positive TSH‐receptor antibodies
February 2019	Referred to endocrinology and commenced carbimazole 10 mg twice daily
2019–2021	Persistent or recurrent biochemical thyrotoxicosis despite progressive escalation of carbimazole
November 2021	Thyroid eye disease diagnosed following development of left proptosis, lid lag and lid retraction. Bilateral pretibial myxoedema was identified clinically
2021–2022	Thyroid eye disease treated with supratarsal triamcinolone and lubricating eye drops. Pretibial myxoedema managed with emollients and compression. Thyrotoxicosis remained inadequately controlled despite high‐dose carbimazole
November 2022	Carbimazole increased to 25 mg three times daily. Definitive treatment discussed because of refractory disease and established thyroid eye disease
January 2023	Perioperative Lugol’s iodine administered, followed by uncomplicated total thyroidectomy. Histopathology showed diffuse hyperplastic changes consistent with Graves’ disease. Levothyroxine commenced postoperatively
June 2024–February 2026	Biochemically euthyroid on levothyroxine. TRAb remained markedly elevated, while thyroid eye disease and pretibial myxoedema remained clinically stable

In November 2021, following a period without in‐person clinical review due to the COVID‐19 pandemic, the patient was found to have developed left‐sided proptosis, lid lag and retraction and was referred to the ophthalmology clinic, whereupon she was diagnosed with TED which was managed with supratarsal triamcinolone and eye drops. This has remained quiescent with a clinical activity score (CAS) persistently less than 2.

During the same review, she exhibited indurated, non‐pitting, erythematous plaques over the pretibial areas bilaterally with a peau d’orange appearance, consistent with PTM (Figure 1). Dermatology was consulted and confirmed the diagnosis clinically. Management comprised emollients, compression and observation; and the condition has remained relatively stable without more invasive therapies.

**Figure 1:**
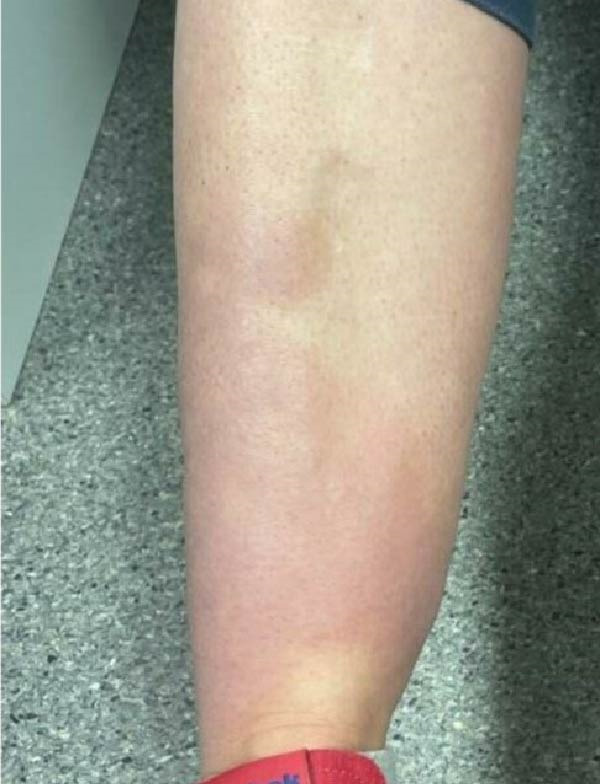
Right pretibial region with erythematous, indurated and non‐pitting dermopathy characteristic of pretibial myxoedema.

CBZ dosage was progressively escalated in response to persistent biochemical thyrotoxicosis, reaching a maximum of 25 mg three times daily. Despite approximately 18 months of high‐dose therapy (≥20 mg twice daily) and good adherence, thyroid function remained poorly controlled, with persistently suppressed TSH and elevated free thyroid hormone levels (Table 1). In the context of refractory disease and the presence of established TED—which made radioiodine less desirable because of the potential risk of exacerbation—a multidisciplinary decision was made to proceed to total thyroidectomy. The patient received perioperative Lugol’s iodine to reduce thyroid vascularity and underwent uncomplicated surgery in January 2023. Histopathology demonstrated diffuse hyperplastic changes consistent with Graves’ disease.

Levothyroxine 125 mcg once daily was initiated post‐operatively. She remains under the care of neurology and endocrinology with persistently raised TRAb titres (Table 1) but with stable TED and PTM. This case illustrates AIGD complicated by both TED and PTM. In randomised trials and real‐world cohorts, 30%–40% of alemtuzumab‐treated patients developed alemtuzumab‐induced thyroid disease (AITD), most commonly AIGD, with a peak incidence between 12 and 36 months after dosing [1, 5].

## 3. Discussion

Alemtuzumab, an anti‐CD52 monoclonal antibody, causes abrupt B and T lymphocyte depletion followed by repopulation from surviving clones, thymic output and homeostatic proliferation in a process known as ‘immune reconstitution’ (IR) [6]. The precise mechanism by which this results in AIGD has not been fully elucidated. Clinically, AIGD can exhibit fluctuations, switching between hyper‐ and hypothyroid states in up to 40% of patients [4] and can be refractory to antithyroid drugs (ATD), requiring prolonged treatment [7]. Surgery or radioiodine may be required when medical therapy fails or is poorly tolerated [5, 7]. Interestingly, the prevalence of AITD among patients treated with alemtuzumab for other autoimmune disorders such as rheumatoid arthritis is much lower, suggesting interplay with the pathophysiological mechanism of MS [2].

Extrathyroidal manifestations in AIGD are uncommon. In a large cohort study of patients with AIGD, the prevalence of TED was 13.4% [3] (compared with 30%–50% in ‘conventional’ GD [8]). A recent review found just 44 cases of AIGD‐associated TED reported in the literature [2]. The presence of TED further influenced management decisions. Radioiodine therapy is generally avoided in patients with orbitopathy due to the risk of exacerbation, even when prophylactic glucocorticoids are used [9]. In this context and given the severity of thyrotoxicosis and inadequate response to medical therapy, total thyroidectomy was considered the most appropriate definitive treatment. Thyroidectomy for alemtuzumab‐related thyroid disease is uncommon. In the largest detailed cohort to date, 5 of 248 alemtuzumab‐treated patients (2.0%) underwent TT, equivalent to 5 of 52 Graves’ disease cases with follow‐up (9.6%) [4].

The patient in this case also developed PTM, a rare extrathyroidal manifestation of Graves’ disease, reported in approximately 0.5%–4% of cases and strongly associated with orbitopathy [9–12]. In two large cohort studies from the UK (*n* = 102) and Norway (*n* = 75), involving patients with AITD, there were no cases of PTM reported [3, 4]. There is one case report in the literature of ‘pretibial oedema’ in a patient treated with alemtuzumab in whom the thyroid function does not appear to have been tested [13]. Pathogenesis of PTM involves TRAb‐mediated activation of TSH‐receptor–expressing fibroblasts and pre‐adipocytes in the skin, with downstream T‐cell cytokines (e.g., IFN‐γ and TNF‐α) promoting marked glycosaminoglycan accumulation within the dermis and subcutis, leading to the characteristic non‐pitting oedema and peau d’orange changes [10, 11]. Although not relevant in our case, smoking is a recognised risk factor and exacerbates both orbitopathy and dermopathy [9], and patients should be counselled to desist.

The clinical spectrum of PTM ranges from subtle plaques to nodular and elephantiasic forms. Most cases are mild and are managed with topical measures and compression. Randomised evidence is limited; case series support intralesional or potent topical corticosteroids under occlusion for symptomatic or progressive lesions [10]. Importantly, PTM can persist (as in this case) or appear despite definitive thyroid ablation. Persistently elevated TRAb titres in this case likely reflect ongoing autoimmune activity driven by IR rather than thyroid antigen availability and have implications for long‐term monitoring for extrathyroidal manifestations of AIGD.

Screening and surveillance are central to safe alemtuzumab use. Consensus guidance recommends thyroid function testing every 3 months for at least 48 months after the last infusion, with prompt evaluation of symptoms suggestive of thyrotoxicosis or hypothyroidism [1, 5]. Serology aids in the diagnosis of Graves’ thyrotoxicosis in this setting. Management follows standard Graves’ pathways but should consider the IR context: ATD responsiveness may be unpredictable and relapse common. Our patient required TT for refractory disease, a strategy supported for severe or unstable AIGD, especially if orbitopathy is present [7].

Patient education on symptom recognition and the potential delayed onset of autoimmune manifestations remains critical. This report is limited by its retrospective nature, interruptions to in‐person assessment during the COVID‐19 pandemic and changes in TRAb assays during follow‐up, which prevent direct quantitative comparison of all antibody results. Nevertheless, the longitudinal clinical, biochemical and photographic findings demonstrate an uncommon combination of refractory AIGD, TED and PTM.

## 4. Conclusion

AIGD can be severe, refractory to antithyroid medication and complicated by extrathyroidal manifestations such as TED and PTM. Pathogenesis reflects dysregulated IR and TRAb‐driven fibroblast activation. Sustained, structured surveillance and multidisciplinary management are required to detect and treat endocrine and dermal sequelae promptly.

## Author Contributions

**Aongus O’Brolchain:** conceptualisation, investigation, data curation, manuscript writing, review and editing. **Elham Reda:** investigation, supervision.

## Funding

This work received no specific funding. Open access publishing facilitated by Griffith University, as part of the Wiley ‐ Griffith University agreement via the Council of Australasian University Librarians.

## Disclosure

All authors have read and approved the final version of the manuscript. Aongus O’Brolchain had full access to all of the data in this report and takes complete responsibility for the integrity of the data and the accuracy of the data analysis.

## Consent

Written informed consent was obtained from the patient for publication of the clinical details and accompanying clinical images.

## Conflicts of Interest

The authors declare no conflicts of interest.

## Data Availability

The data are available upon reasonable request from the authors.
